# A Qualitative Phenomenological Study of Treatment Options for Patients with SCD and Chronic Pain: Buprenorphine–Naloxone or Full-Agonist Therapy

**DOI:** 10.3390/ijerph23060813

**Published:** 2026-06-18

**Authors:** Najibah Galadanci, Calia Torres, Terrika Johnson, Julie Kanter

**Affiliations:** 1 Lifespan Comprehensive Sickle Cell Center, Department of Medicine, Division of Hematology and Oncology, University of Alabama at Birmingham, Birmingham, AL 35294, USA; ngaladanci@uabmc.edu (N.G.); terrikajohnson@uabmc.edu (T.J.); 2 Gulfshore Behavioral Health, Fort Myers, FL 33907, USA; caliatorres@gmail.com

**Keywords:** sickle cell disease, chronic pain, buprenorphine-naloxone, qualitative research, pain management, quality of life

## Abstract

**Highlights:**

**Public health relevance—How does this work relate to a public health issue?**

- Chronic pain in sickle cell disease (SCD) is common and contributes to high healthcare utilization, reduced quality of life, and persistent health disparities.
- This study explores patient experiences with chronic opioid therapy and buprenorphine–naloxone (bup-nal) as alternative approaches to managing chronic pain in SCD.

**Public health significance—Why is this work of significance to public health?**

- Findings highlight a gap between current pain management strategies and patient priorities, particularly the importance of daily functioning and stability.
- The study provides patient-centered insights into pain management in a population disproportionately affected by structural inequities and stigma.

**Public health implications—What are the key implications or messages?**

- Practitioners should engage patients in shared decision-making regarding chronic pain management and clearly discuss the potential benefits and limitations of buprenorphine-naloxone, particularly with respect to chronic versus acute pain.
- Policymakers and researchers should prioritize strategies that improve access to comprehensive, patient-centered pain management while addressing stigma and evaluating outcomes such as functioning, quality of life, and healthcare utilization.

**Abstract:**

Data in sickle cell disease (SCD) shows that 30–50% of adults have chronic pain. Chronic pain is a life-shaping condition that is often inadequately managed with chronic opioid therapy (COT). Buprenorphine–naloxone (bup-nal) is an alternative (to COT), yet patient perspectives on its use remain limited. This is a phenomenological qualitative study exploring how adults with SCD experience chronic pain and consider treatment options including COT and bup-nal. Semi-structured interviews were conducted with 26 adults with SCD and chronic pain who were offered pain management with bup-nal, including individuals who declined or discontinued treatment. Participants described pain as a constant, embodied experience around which they structured their daily functioning, relationships, well-being, and treatment. Those receiving bup-nal consistently reported improved daily functioning, greater independence, enhanced mood, and reduced healthcare utilization. In contrast, participants not receiving bup-nal described ongoing pain interference, reduced daily functioning, and continued reliance on acute care services. Importantly, participants emphasized functional improvement and stability, rather than pain elimination, as the most meaningful outcomes. These findings suggest that buprenorphine–naloxone may shift pain from a dominant, disabling experience to a more manageable condition that improves participation in work, relationships, and daily activities. To optimize management of chronic pain in SCD, it is necessary to align treatment with patient priorities.

## 1. Introduction

Sickle cell disease (SCD) is a chronic, multisystem disorder characterized by vaso-occlusion, hemolysis and progressive organ damage that contribute to substantial morbidity, impaired health-related quality of life, and high healthcare utilization [1,2].

Pain is the hallmark manifestation of SCD and the most common reason individuals seek medical care [3]. While vaso-occlusive pain episodes are a hallmark manifestation of SCD, 30–50% of adults also experience chronic pain arising from multifactorial mechanisms including recurrent tissue injury, inflammation, neuropathic processes, and cumulative organ damage [4]. For many affected persons, severe pain episodes occur unpredictably and coexist with ongoing chronic pain, making coping difficult and socially disruptive. As individuals survive into adulthood, chronic pain becomes a lived, embodied experience that affects emotional well-being, identity, employment, relationships, and participation in daily activities [5,6]. Despite this, clinical pain management strategies have often prioritized acute crisis treatment over the longitudinal experience of daily pain [7].

Opioid analgesics remain the mainstay of treatment for acute pain crisis in SCD and are guideline-recommended for this indication [8]. However, chronic opioid therapy (COT) is not routinely recommended for chronic pain due to concerns around limited effectiveness, tolerance, opioid-induced hyperalgesia, and adverse effects that can impair functioning [9,10]. Further, some adolescents and adults with SCD experience persistent, refractory pain despite COT, often accompanied by frequent emergency department visits and hospitalizations [11,12]. These patterns of care are associated with diminished quality of life, increased risk of early mortality, and heightened exposure to stigma within healthcare settings. Recent quantitative studies have also demonstrated that implementation of broader opioid prescribing policies, including the CDC opioid prescribing guidelines, was associated with reductions in opioid prescribing among individuals with SCD despite the unique pain management needs of this population. These findings have raised concerns that efforts to address the opioid epidemic may unintentionally create additional barriers to effective pain management for people living with SCD [13]. These challenges are further compounded by structural and racial inequities. In the United States, where SCD predominantly affects Black individuals, patients with SCD are disproportionately perceived as being at increased risk for opioid misuse and are more likely to experience heightened monitoring, stigma, and referral to substance use services when receiving opioids for pain [14,15,16]. As a result, individuals with SCD often encounter unique barriers to effective pain management in the acute care environment and healthcare system in general, including mistrust, moral judgment, and constrained treatment options, which shapes their engagement with the healthcare systems [16,17].

Buprenorphine–naloxone (bup-nal), a partial opioid agonist medication marketed under the brand name Suboxone among others, has emerged as a potential alternative approach for managing chronic SCD pain. Bup-nal is a combination medication consisting of buprenorphine, a partial μ-opioid receptor agonist, and naloxone, an opioid antagonist included primarily to deter injection misuse [18,19,20]. Compared with full opioid agonists, buprenorphine demonstrates a ceiling effect on respiratory depression and may provide more stable analgesia with lower risk of tolerance and opioid-induced hyperalgesia, making it a potential alternative for chronic pain management [21,22]. In current clinical practice, bup-nal is typically considered for individuals with chronic pain who have experienced inadequate benefit, unacceptable adverse effects, or escalating opioid requirements while receiving chronic opioid therapy [22].

Emerging evidence suggests that, when used in conjunction with disease-modifying therapies in specialized SCD care settings, bup-nal is safe, well tolerated, and associated with reduced acute healthcare utilization [23,24,25]. In a retrospective study, adults who transitioned from full agonist COT to bup-nal experienced substantial reductions in hospital admissions, along with reported improvements in sleep, mood, and overall quality of life [23]. Similar findings have been observed in real-world, including the use of the “David–Carroll protocol,” where patients described functional improvements within weeks of transition [26].

Despite these promising findings, bup-nal remains more commonly associated with treatment for opioid use disorder [27], which may contribute to hesitation and concern among patients with SCD [28]. In addition, misalignment between patient expectations for pain relief particularly during acute vaso-occlusive crises and the pharmacologic profile of bup-nal further complicates decision-making [23,24]. As a result, some individuals decline or discontinue bup-nal despite potential benefits for daily stability and functioning [28].

**Figure 1:**
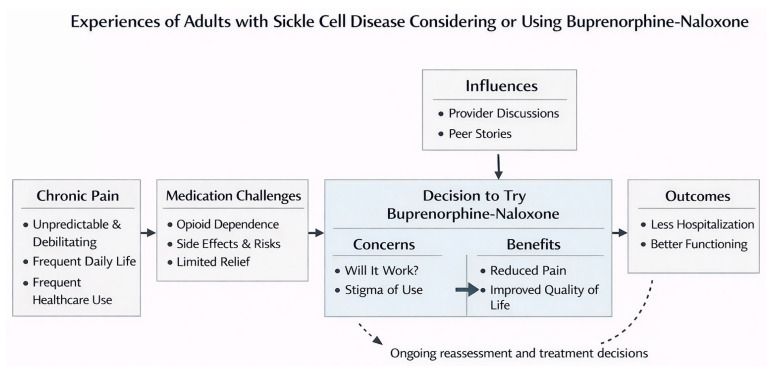
Theoretical framework describing experiences of adults with sickle cell disease considering or using Buprenorphine–Naloxone.

To date, limited research has explored how adults with SCD understand and experience chronic pain, risks and benefits of COT, and the decision to initiate, continue, or decline bup-nal. Understanding these lived experiences is essential to informing patient-centered pain management strategies that prioritize function, quality of life, and alignment with patient goals. The aim of this qualitative study was to explore the perspectives of adults with SCD and chronic pain regarding their lived experience of pain, the impact of pain on daily life, experiences with opioid therapy, and perceived barriers and facilitators to using bup-nal within routine clinical care. The conceptual framework guiding this study is summarized in Figure 1.

## 2. Materials and Methods

### 2.1. Study Design, Sample and Setting

This qualitative study used a phenomenological approach [29] to explore the lived experiences of adults with SCD related to chronic pain, opioid therapy, and the use or consideration of bup-nal in daily life. A phenomenological design was selected to capture the depth and complexity of participants’ subjective experiences, allowing for a rich understanding of how they perceive, interpret, and make meaning of pain, treatment, and quality of life [29,30]. This approach is particularly suited for SCD, where individual experiences are shaped by biological, emotional, and social factors, as well as stigma and healthcare interactions. By focusing on participants’ own narratives, the study sought to identify common meanings and variations in how individuals understand their pain, medication concerns, and decisions regarding bup-nal treatment. The use of an inductive, interpretive method ensured that findings were grounded in participants’ perspectives rather than pre-existing theoretical assumptions, consistent with phenomenological inquiry aimed at describing the essence of lived experience.

Purposive sampling was used to recruit adult participants with SCD who had experience with chronic pain and were offered bup-nal for pain management. Participants were recruited between August 2024 and November 2024 from the Lifespan Comprehensive SCD center at the University of Alabama at Birmingham, Birmingham, Alabama, United States. Eligibility criteria included: (1) age ≥ 18 years; (2) confirmed diagnosis of SCD; (3) experience of chronic pain, defined as pain occurring on most days for at least three months; (4) a history of opioid use for pain management; (5) ability to speak and understand English; and (6) either current use of bup-nal, prior use and discontinued, or having declined bup-nal after discussion with a healthcare provider. Participants were receiving standard SCD-related therapies when clinically indicated, including hydroxyurea, transfusion therapy, and/or other disease-modifying therapies.

Recruitment occurred through direct referral by the clinical team. A total of 28 adults were approached and all agreed to participate. We were successfully able to interview 26 patients (the remaining 2 participants could not be reached to schedule the interview prior to data saturation). Our initial sampling strategy aimed to recruit approximately equal numbers of participants receiving and not receiving bup-nal to ensure representation of diverse treatment experiences. However, qualitative recruitment was guided by data saturation rather than a predetermined subgroup sample size. As recruitment progressed, a larger proportion of eligible participants were actively receiving bup-nal, resulting in a final sample of 16 participants receiving bup-nal and 10 participants who had declined or discontinued treatment. Sample size was ultimately determined by data saturation, defined as the point at which no new information or themes were forthcoming.

### 2.2. Data Collection

Ethical approval to conduct the study was obtained from the University of Alabama at Birmingham Institutional Review Board (IRB) prior to data collection. Semi-structured, in-depth interviews were developed by N.G and J.K to explore perspectives of adults living with SCD diagnosed with chronic pain on their pain experience, pain interference, quality of life, concerns and challenges related to pain medication and barriers and facilitators to using bup-nal. An interview guide was developed based on prior literature on SCD pain, COT, emerging evidence on bup-nal use in SCD, and the research team’s clinical and qualitative expertise. Probes were used to elicit rich descriptions and clarify participant responses. Participants’ answers to initial questions guided subsequent questioning. Participants were read the full verbal consent statement approved by the institutional review board and verbally agreed to participate prior to initiation of the interview. Interviews were conducted by N.G, a female hematologist and epidemiologist (PhD) with extensive training and experience in qualitative research. The Interviewer had no prior relationship with the participants prior to the interview. At the beginning of the interview, N.G introduced herself, her background and her role in the study. Demographic data were collected from each interview participant and included age, race/ethnicity, gender, educational level and employment.

Interviews were conducted using Zoom Workplace (version: 6.6.10 (69071)) and audio recorded. All interviews were conducted individually in a one-on-one format between the participant and the interviewer, no group or focus-group interviews were performed. Participants were encouraged to be in a private location during interviews, and the interviewer was always in a private location. Interviews lasted 20–58 (mean 39) min. Field notes were written during and immediately following interviews to capture contextual details and analytic reflections. Participants received a gift card as compensation for their time. Audio files were sent to an external, Health Insurance Portability and Accountability Act (HIPAA)-compliant agency for transcription, and transcribed interviews were reviewed for quality and accuracy, and redacted for identifiable information by N.G. No repeat interviews were conducted, and transcripts were not returned to participants for correction or comment. Data saturation was reached at 26 interviews (16 participants currently on bup-nal, and 10 participants not on bup-nal). The conduct and reporting of this study followed the COnsolidated criteria for REporting Qualitative research (COREQ) checklist [31].

### 2.3. Data Analysis

Data were analyzed using an interpretive phenomenological approach, with a focus on understanding how participants made meaning of chronic pain, opioid therapy, and how they considered bup-nal for chronic pain. Data collection and analysis occurred concurrently, allowing early insights to inform subsequent interviews.

All transcripts were de-identified and assigned unique participant identification numbers. N.G and C.T independently read each transcript in its entirety to achieve immersion in the data. Transcripts were analyzed using NVIVO 14 software (Lumivero, Denver, CO, USA). Initial coding was conducted inductively, with both coders identifying significant statements and meaning units related to pain experiences, medication use, and daily functioning. Following independent coding of an initial subset of transcripts, the coders met to compare codes, discuss discrepancies, and refine code definitions. A preliminary codebook was developed through consensus and applied to subsequent transcripts. The two coders continued to meet regularly throughout the analysis process to review coding decisions, resolve disagreements, and ensure consistent application of codes. Intercoder agreement was achieved through discussion and consensus rather than statistical calculation, consistent with qualitative phenomenological methodology.

Codes were grouped into categories and synthesized into themes and subthemes that reflected shared aspects of participants lived experiences. Variations in experiences between participants who continued, discontinued, or declined bup-nal were examined within themes to capture differences in meaning while preserving a unified phenomenological framework. Analysis continued until thematic saturation was reached, defined as the point at which no new themes or substantive variations emerged [32].

### 2.4. Qualitative Rigor

The interviewer (N.G.) is a hematologist and epidemiologist with expertise in SCD and qualitative research. While she is affiliated with the UAB Lifespan comprehensive SCD center where participants received care, she is not involved in the patients’ clinical management. She had no established clinical relationship with participants prior to the interviews. To minimize potential bias, interviews were guided by a semi-structured interview guide, participants were encouraged to discuss both positive and negative experiences with pain management and bup-nal, and coding was conducted independently by two researchers with consensus-based theme development.

Several strategies were employed to ensure rigor and trustworthiness. To ensure credibility, the interviewer asked participants to clarify statements, use verbatim transcripts, and included rich participant quotations followed by iterative engagement with the data by two analysts. Dependability was enhanced through the involvement of two independent coders and ongoing consensus-based discussions to achieve intercoder agreement. The research team’s combined expertise in SCD, pain management, and qualitative research methods further supported the rigor of the analysis. Together, these strategies ensured that the findings were grounded in participants’ accounts and accurately represented their lived experiences.

## 3. Results

### 3.1. Participants Characteristics

The demographic characteristics of the interview participants are presented in Table 1. A total of 26 adult patients with a mean age of ~29 years (IQR: 23–32) reflecting a young adult population living with chronic SCD-related pain. The participants were predominantly female (*n* = 18, 69%) and all identified as Blacks or African Americans. All participants reported chronic pain, a history of COT for pain management and had been offered bup-nal for chronic pain management. At the time of interview, 62% of participants (*n* = 16) were actively taking bup-nal, while 38% (*n* = 10) were not on bup-nal, including individuals who had declined initiation or tried and discontinued the medication. The sample included participants currently receiving bup-nal as well as individuals who had been offered the same therapy (bup-nal) and had declined or discontinued treatment, allowing representation of a range of treatment experiences. Participants receiving bup-nal and those not receiving bup-nal were generally similar in age and educational attainment. Overall, 17 participants (46.2%) reported a high school education, some college education, or current college enrollment, 8 (42.3%) had completed a bachelor’s degree, and 1 (11.5%) had completed a master’s degree. Financial challenges were common in both groups. Nine participants (34.6%) reported receiving disability income, while 12 (46.2%) reported annual incomes below $40,000. Despite this, many participants reported limited income, with over one-third relying on disability income and nearly half reporting annual incomes below $40,000.

### 3.2. Overview of Themes

Using a phenomenological approach, we sought to understand how adults with SCD experience chronic pain, opioid therapy, and decision to initiate, continue, discontinue or decline bup-nal in the context of daily life. Eight phenomenological themes capture the essence of this lived experience. Table 2 provides an overview of the relationship between key analytic codes and the resulting phenomenological themes. Across interviews, participants did not describe pain as an isolated symptom but as an experience that shaped identity, routine, relationships, emotions, and treatment decisions. While participants shared a common experience of persistent, life-shaping pain, those receiving bup-nal consistently described meaningful improvements in daily functioning, emotional well-being, and healthcare stability, whereas those who declined or discontinued bup-nal described lives largely structured around uncontrolled pain, limited function, and ongoing distress. These differences reflect variation not only in treatment choice but in how pain was experienced and managed in everyday life.

#### 3.2.1. Theme 1: SCD Pain Is Experienced as a Distinct, Embodied Phenomenon

Participants described SCD pain as something that was immediately recognizable in their bodies and qualitatively different from other types of pain. This distinctiveness was central to how they understood their illness and assessed the seriousness of symptoms. Although the study focused on chronic pain, participants frequently described acute vaso-occlusive pain when explaining how they recognized SCD-related pain and distinguished it from chronic daily pain. These descriptions provided important context for understanding subsequent discussions regarding chronic pain management and treatment decisions.

##### Subtheme 1a: SCD Pain as Qualitatively Different and Unmistakable

Participants emphasized that they felt pain caused by SCD had a particular quality deep, stabbing, crushing, or burning that felt different from muscle soreness, headache, or injury. They described an immediate, visceral recognition that the pain was distinct, different and more serious. They spoke about knowing when pain was sickle-related without needing to analyze it.

*“It’d be like a constant ache and throbbing type thing normally when I have my crisis. Sharp pain, if it’s in my chest, it hurt to breathe or something like that. If it’s in my joints, it’ll hurt to walk, and it’ll be sharp and stuff like that.”*

Participant-005 (receiving bup-nal)

*“Pins and needles sticking in your joints.”*

Participant-003 (receiving bup-nal)

*“It feels like my bones are being crushed from the inside out.”*

Participant-025 (not receiving bup-nal)

##### Subtheme: SCD Pain Is Experienced with Fear and Emotional Distress

Beyond the physical sensation, participants experienced pain mixed with emotional responses including fear, anxiety and urgency that formed part of their illness identity. They believed that living long enough had taught them to read their own bodies and to include both the physical sensation and heightened awareness that caused them to recognize when it (the pain) needed clinical attention.

*“Just calming’ my mind down because of how anxious I feel whenever I have pain. Even if I don’t have pain, it’s just still that anxiety of I’m always feeling’ like something is off, feeling’ like something is going’ to happen, or whenever it will happen…”*

Participant-002 (receiving bup-nal)

*“I learned how to more so deal with it emotionally as much as possible, but sometimes it can be really hard… and there’s nothing you can really do”*

Participant-023 (not receiving bup-nal)

*“It was a deep depression emotionally because it’s like you can’t do nothing. You wanna do something, but you can’t do nothing’ cause you’re in so much pain…”*

Participant-004 (receiving bup-nal)

#### 3.2.2. Theme 2: Chronic Pain Becomes the Background State of Living

Chronic pain was not described as episodic, or occurring only during crises, rather it was described as a persistent presence that shaped everyday life.

##### Subtheme 2a: The Process of Adapting to Pain

Participants described a process of adaptation to pain rather than improvement. Chronic pain was described as a daily condition of nagging, dull, aching, or burning that they learned to live with. This “normalization” did not mean the pain was mild; rather, it meant participants had adapted to living with constant discomfort.

*“That constant never go away dull, achy pain. That stiffness, that soreness, that just nagging tight pain. That’s just always lurking and always there. That’s that type of pain.”*

Participant-015 (receiving bup-nal)

*“To me, it’s a yo-yo cycle of going’ from trying’ to get better from taking medicine till the medicine wear off, and then you start the process all over”*

Participant-009 (receiving bup-nal)

##### Subtheme: Living with Pain That Becomes Normal

Because pain was constant, participants often describe themselves as fine except during exacerbations. This experience created a paradox: participants were living in pain every day yet often described themselves as fine. Pain only becomes visible or legitimate to the patients when it escalates into a “crises,” reinforcing cycles of delayed care and misunderstanding.

*“It’s like continuous, continuous minutes of pain to hours. It can be hours or days of pain.”*

Participant-001

*“It’s nonstop. It never stops. but it’s not something that you can allow to put your day at a halt because it’s something that is pretty long-term.”*

Participant-025 (not receiving bup-nal)

*“basically just in pain most of the time very excruciating. Just in pain all the time chronically. Something you just get used to”*

Participant-017 (receiving bup-nal)

#### 3.2.3. Theme 3: Pain Structures Daily Life, Roles, and Relationships

Pain shaped not only physical functioning but also how participants planned, interacted and fulfilled social roles.

##### Subtheme 3a: Planning Life Around Pain

Participants described organizing their days around anticipated pain. Decisions about work, social events, travel, and family responsibilities were filtered through the question: How much pain will I be in? While some opted to modify their activity levels and involvement in certain activities, others found the pain was debilitating and their concern for potential pain exacerbation led them to avoid activities.

*“You are trying to schedule your day around how you’re gonna feel and how much pain you think you’re gonna be in. You’re scheduling what you’re trying’ to do about what you need to do for the day and what you can do for the day ’cause of how much pain you think is going to.”*

Participant-009 (receiving bup-nal)

*“I schedule my day around how much pain I think I’m gonna be in.”*

Participant-004 (receiving bup-nal)

*“If I have plans or if I want to do something, out of nowhere, it’ll come, and I will have to cancel those plans. Sometimes, I have to miss work because it can be times where I can’t even get out the bed for a couple hours, I can’t keep a job because of the pain.”*

Participant-001 (receiving bup-nal)

##### Subtheme 3b: Social Disruption, Guilt and Emotional Burden

Patients with SCD felt that pain impacted more than their physical functioning. Pain led to cancelled plans, missed work, and withdrawal from relationships leading to guilt, frustration and emotional distress. Several participants expressed guilt about not being able to fulfill expected roles as parents, partners, or employees. Others described isolation because friends and family did not understand why plans were frequently cancelled. Pain was therefore not only physical; it was socially disruptive, reshaping how participants engaged with the world.

*“That’s what I felt. Very, very frustrated, very, very tired. Sometimes it was depressing. Sometimes it’s lonely because not everyone understands what you’re goin’ through. Even people who live with you and know you all of your life, they can only empathize for—no one really gets it all the time.”*

Participant-009 (receiving bup-nal)

*“That’s why I get so angry about stuff. I do have a house. I do own a house. I do own a car. I do have kids. If it come to where I cannot work, and I don’t work, I don’t get paid, in order to take care of my bills. Because the power company doesn’t care that you have sickle cell, and you in pain, and you can’t move, …”*

Participant-007 (receiving bup-nal)

*“It affect my life a big way from relationship, meaning that couldn’t really go on dates. As a man, it was hard, … I felt less than a man, I felt weak. It made me depressed because I couldn’t do what I needed to do as a man like provide for my family, even provide for myself. As a man you don’t really have a lotta handouts, so it was frustrating, it was depression. It was me not being able to go out on dates, not being able to go hang out with my friends. I had to cancel dates.”*

Participant-008 (receiving bup-nal)

*“When I have pain, I feel sad, and it do make me depressed at time because it’s like, when I’m in pain, I’m missing out on other stuff that I could be doing in life, but instead, I can’t because of the pain.”*

Participant-020 (not receiving bup-nal)

#### 3.2.4. Theme 4: Chronic Opioid Therapy as an Exhausting Search for Pain Relief That Never Fully Works

Participants described the ongoing use of chronic opioid therapy as an attempt to control pain marked by uncertainty regarding treatment effectiveness, frustration with persistent pain, and dissatisfaction with long-term outcomes. Across narratives, opioids were framed as the only available option but not as a “desired treatment.” Participants emphasized that their primary goal was pain relief irrespective of the type of pain. Over time, this uncertainty, combined with side effects and limited effectiveness, contributed to dissatisfaction and a desire to avoid chronic opioid use.

##### Subtheme 4a: Seeking Pain Relief, but Unclear Which Pain Is Being Treated

Participants described confusion about whether opioids were meant to treat acute crisis pain (from SCD), chronic daily pain, or both. Many reported continuing opioids because pain was present, even when they were unsure whether the medication was effective at targeting the pain. This lack of clarity contributed to ongoing medication use despite limited benefit.

*“It works… but then it doesn’t because it’s just so temporary that the pain is you’re going to eventually going to be back in pain. It just I don’t know I guess it don’t help basically.”*

Participant-017 (receiving bup-nal)

For some, opioids dulled pain temporarily without addressing the underlying experience of constant discomfort, leaving them unsure whether the medication was truly helping.

##### Subtheme 4b: Temporary Relief, Tolerance and Side Effects

Participants described a pattern of pain experience in which opioids initially provided relief but became less effective over time. As COT became less effective, higher doses were required, yet pain usually persisted. In addition to limited effectiveness, participants described difficulty tolerating COT due to side effects such as sedation, mental fog, and interference with daily activities. Daily routines are often organized around medication timing and recovery from its effects. This cycle was described as physically and emotionally exhausting.

*“It’s a yo-yo cycle when the medicine wear off. This is just my experience. I have this pain medication. To me, it works for me, but it only works for a certain amount of time. I’m like, ‘Okay, it’s only 10 milligrams. Lemme take this one.’ You get up. You take your one. Then you see, an hour, maybe to an hour and 30 min to 2 h, it’s not workin.”*

Participant-004 (receiving bup-nal)

*“You name it, I was on it… fentanyl patches, oxycodone, all of it. From Percocet, Methadone, Fentanyl patches, Oxycodone, Oxycontin and all that. I’ve been on a list of opioids over my 29 years on this world. Even starting at a young age, I’m having Tylenol with Codeine and moving on up to Percocet tabs and all the way up to Fentanyl patches and stuff like that, so I was on a lot of opioids. I wasn’t even a big guy; I was still 130 pounds soaking wet, and I was on these heavy medications.”*

Participant-008 (receiving bup-nal)

*“I was getting stronger doses and stronger doses and stronger doses so my body could fight off the pain I was getting. The more you take em, the more your body get used to them… and that’s not doing anything anymore for the pain.”*

Participant-009 (receiving bup-nal)

Participants emphasized that they were not seeking euphoria; they wanted to function. Instead, opioids often left them feeling impaired and disconnected from daily life.

##### Subtheme 4c: Frustration and Desire to Avoid Chronic Opioid Use

A strong sense of frustration emerged as participants reflected on years of opioid use without sustained relief. Many described being tired of taking increasing amounts of medication without meaningful improvement and expressed a desire to find alternatives.

*“The reason I wanted to get on Suboxone was because I felt like I was taking too many of the Norcos, and I just felt like… and I was just tired of having pains.”*

Participant-007 (receiving bup-nal)

For many, opioids came to represent a treatment they endured rather than a choice. It was something that felt unavoidable but unsustainable.

#### 3.2.5. Theme 5: Stigma and Moral Judgment Shape Medication Experiences

Participants described stigma as strong force influencing how pain treatment was experienced and discussed.

##### Subtheme 5a: Feeling Judged and Misunderstood by Others

Participants felt labeled as “drug-seeking” by providers and misunderstood by family members. These judgements affected how openly they discussed pain and medication needs and contributed to feelings of shame and frustration. The association of bup-nal with opioid use disorder or addiction intensified these feelings and made individuals question their need for this management. Several participants were concerned that taking bup-nal would label them as “addicts” both by their healthcare team as well as by family and society. They needed reassurance that the medication was not being used for addiction treatment.

*“I don’t even know where to go with it because it’s kind of annoying. Because being on opioids as a sickle cell patient of course make doctors…, look at you like you’re a druggie or a crackhead or just in there for a hit.”*

Participant-019 (not receiving bup-nal)

*“Family think we just want drugs.”*

Participant-018 (not receiving bup-nal)

*“My family don’t understand why I need medication”*

Participant-014 (receiving bup-nal)

*“Sometimes our own family members will make us feel like we’re drug addicts because we just want the pain to stop.”*

Participant-010 (receiving bup-nal)

##### Subtheme 5b: Invalidation and Loss of Trust

Participants often felt invalidated and ignored by healthcare providers due to repeated experiences of dismissal regarding their pain. The feeling of being dismissed contributed to a general mistrust in the healthcare establishment and reinforced feelings of marginalization that also transferred to their concerns around bup-nal.

*“I feel really invalidated and really ignored when I’m saying that I’m hurting, and they’re like, ‘Oh. Well, it’s chronic pain. Just take Tylenol,’ and I’ve taken enough medicine to tranquilize a horse, and they think that Tylenol will be like the deciding factor. I think that some changes need to be made in that regard.”*

Participant-025 (not receiving bup-nal)

#### 3.2.6. Theme 6: Starting Bup-Nal as a Self-Directed Decision Informed by Clinical Guidance

Many participants described initiating bup-nal after it was introduced or recommended by their healthcare provider. For some, this was their first exposure to the medication, while others had previously heard of it but had limited understanding of how it worked.

##### Subtheme 6a: Careful Decision Making

Although provider recommendation for pain management with bup-nal often prompted consideration, participants emphasized that the decision to start therapy was their own. Participants engaged in thoughtful deliberation before starting treatment. They asked questions about potential side effects, how the medication would make them feel on a daily basis, whether it would effectively prevent withdrawal, and how other patients (with SCD) had responded to it.

*“No, I really didn’t talk to a lotta people before I started the suboxone. It was just my primary care provider, and we talked about it.”*

Participant-011 (receiving bup-nal)

*“I wanted to know how it would affect me every day.”*

Participant-006

*“I asked if other patients said it worked.”*

Participant-006 (receiving bup-nal)

*“I decided on my own because I just was fed up of, like I said That’s cause when you talk to other sickle cell patients, they’ll be like, it’s okay. It’s all right.”*

Participant-005 (receiving bup-nal)

##### Subtheme 6b: Navigating Family Reactions

Most participants reported not discussing the decision with family members, often due to concerns that they would not understand or worse, further stigmatize them. A few participants did feel they could approach family members and (in those cases) found they were supportive and encouraged them to trial the bup-nal and to see if they would feel better.

*“I did talk to somebody, but I didn’t have to. I told them what I was going to do, but I didn’t have to get anyone’s permission. No, no. I think everybody was—my family was over me and the pills.”*

Participant-009 (receiving bup-nal)

*“Well I talked to my mother cause she is a nurse. I kinda talked to my mother about it. We knew very little about it. They kinda told us don’t read what was on the internet about the medication because they was using it for other reasons besides what was on the internet about the medication.”*

Participant-007 (receiving bup-nal)

#### 3.2.7. Theme 7: Bup-Nal Reshapes Daily Life by Changing How Pain Is Lived, Managed, and Tolerated

Across interviews, participants who opted to take bup-nal described the medication primarily in terms of its effect on daily life. For those who continued bup-nal, its benefit was described through changes in activity, emotional wellbeing, independence, relationships, and healthcare use. For those who stopped or declined bup-nal, dissatisfaction centered on the medication’s inability to meet expectations for pain relief particularly during acute pain crises or due to emotional or physical side effects that outweighed perceived benefits. Together, these narratives reveal that bup-nal was experienced as a treatment that meaningfully improved daily life for many participants, shifting pain from a dominant, disabling force to a more manageable background condition. In contrast, participants not on bup-nal described continued high levels of pain interference, limited functioning, and ongoing emotional distress.

##### Subtheme 7a: Daily Activities and Independence Become Possible but Not Unlimited

Many participants who remained on bup-nal described a meaningful increase in their ability to engage in everyday activities. They emphasized being able to get out of bed, complete household tasks, work, attend school, care for children, and participate in previously avoided physical activities. Importantly, participants did not describe becoming “pain-free,” but rather described a reduction in pain interference.

*“I wake up and I don’t think about pain. I’m not in pain every day. I’m able to do more… I work, I’m in school, and I’m able to be a mom to my child.”*

Participant-013 (receiving bup-nal)

*“Now I don’t have that everyday pain.”*

Participant-017 (receiving bup-nal)

*“I’m not sick in bed all day… I’m back to being, for the most part, my usual self.”*

Participant-009 (receiving bup-nal)

*“I’m not hurting every day either. You know what I’m saying? That allows me to do whatever that I can do, and I’m not sick in bed all day.”*

Participant-005 (receiving bup-nal)

*“I guess, now when I’m in pain, I’m only in pain at night or under unusual circumstances. Maybe I just didn’t get enough sleep, or I’m not just feeling too good that day, but I have noticeably more energy, … He came to my apartment, and I was running up and down the steps. I live three stories up. He took note of that. He’s like, Yeah, you definitely weren’t doin’ that before.”*

Participant-012 (receiving bup-nal)

*“Really, I wake up and I don’t think about pain. I’m not in pain every day. I’m able to do more. As I said, I work, I’m in school, so that was a good success for me, and I’m able to be a mom to my child.”*

Participant-013 (receiving bup-nal)

##### Subtheme 7b: Emotional Impacts of Bup-Nal Treatment

Participants frequently described emotional and mental changes as central to their experience on bup-nal. They noted that the reduction in daily pain translated into improved mood, decreased depression, and renewed motivation to engage with life. Several participants aptly compared this emotional state to their prior experiences of isolation, sadness, or emotional numbness when pain dominated their days.

*“I feel a lot better. I’m not down… I have energy now. I can get up and do my daily activities. I can go to work, and I can also take my medicine while being at work and still be able to get through the day.”*

Participant-001 (receiving bup-nal)

*“Mentally, I’m not depressed… I don’t stay in my room a lot. I’m able to get up, enjoy life mentally. I’m not as stressed out because I’m able to provide. Suboxone gave me the opportunity to be able to go to work and provide for my family, so mentally and physically, it’s better. My family even said it; they are proud of me cause I haven’t been in the hospital for four years I’m able to get up and enjoy life.”*

Participant-008 (receiving bup-nal)

*“I feel better emotionally and mentally. My mood is a lot better. I’m not down and depressed, always thinking bad things”*

Participant-013 (receiving bup-nal)

However, not all emotional effects (of bup-nal) were positive. Participants who stopped bup-nal or felt it was ineffective described emotional worsening tied to ongoing pain, disappointment, or feeling that the medication failed to meet their needs.

*“I feel worse, honestly… I started getting more depressed because I was in so much pain all the time.”*

Participant-014 (receiving bup-nal)

##### Subtheme 7c: Reduced Hospitalizations as a Marker of Success

A dominant marker of bup-nal’s effectiveness for many participants was a dramatic reduction in emergency department visits and hospital admissions. Participants repeatedly discussed fewer hospitalizations as evidence that their pain was more manageable and their lives more stable.

*“Since I been on it, I was finally able to go a whole year without actually being admitted into the hospital. I don’t have to go to the pain clinic as often as I used to, and it’s just—it makes me be able to be more free and be able to actually enjoy every day, basically”*

Participant-003 (receiving bup-nal)

*“For three years, I’ve never been admitted for sickle cell pain.”*

Participant-008 (receiving bup-nal)

*“I have not had any hospital visits. I haven’t had any ER visits. I feel like it’s good. My pain is really well controlled now. I’m not gonna say it’s a cure for sickle cell, but it felt like a cure for sickle cell for me.”*

Participant-004 (receiving bup-nal)

For these individuals, staying out of the hospital allowed them to be more socially active, being present for holidays, work, and family milestones that had previously been interrupted by admissions.

##### Subtheme 7d: While Chronic Pain Improves, Acute Crisis Pain Remains a Concern

Participants consistently distinguished between daily chronic pain and acute pain crisis when describing the effect of bup-nal. Many patients reported substantial improvements in daily pain while simultaneously expressing concern that bup-nal did not provide sufficient relief during severe crises.

*“It helps with the everyday pain… but when I finally did have a crisis, it felt like it wasn’t helping.”*

Participant-005 (receiving bup-nal)

*“Great when I’m not in that much pain. I don’t think it is a good medicine for when you’re in a terrible bad pain crisis to stop the pain”*

Participant-017 (not receiving bup-nal)

*“I stopped taking it because for one, I didn’t really feel like it was helping. Then it was just like I don’t know. I felt like if it… I didn’t feel like it was helping”*

Participant-021 (not receiving bup-nal)

This distinction was central to why some participants discontinued or declined bup-nal. For them, the inability to rapidly relieve intense crisis pain outweighed gains in daily stability.

##### Subtheme 7e: Relationships and Social Re-Engagement

Participants who experienced benefit from bup-nal described improved relationships and increased social participation. The reduction in pain allowed them to spend time with family, maintain employment, and reduce reliance on others for daily needs.

*“I’m not having to depend on my family to do the mundane things.”*

Participant-009 (receiving bup-nal)

*“My family even said it. They’re proud of me.”*

Participant-008 (receiving bup-nal)

Conversely, participants who did not benefit (or did not try bup-nal) described continued withdrawal, limited interaction, and emotional numbness driven by persistent pain.

*“I’m thinking it was just the emotional, what it was doing for me. I think it was with what it was affecting me mentally or something like that. Like I knew I had to take it, but… I think it was higher than what I needed. I think that probably was like, yeah, I don’t want to take it no more cause I don’t—maybe it was just too much for my system”.*

Participant-023 (not receiving bup-nal)

*“It helped with the pain, it’s just it was very [distorted audio 06:44] just was very uncomfortable”*

Participant-024 (not receiving bup-nal)

Across subthemes, a consistent pattern emerged: participants on bup-nal described regained function, improved mood, and reduced healthcare utilization, whereas those not on bup-nal continued to experience persistent limitations, emotional burden, and frequent disruption of daily life due to pain.

#### 3.2.8. Theme 8: When Bup-Nal Does Not Align with Expectations, Pain Remains Central and Unresolved

Participants who declined, discontinued, or felt unsure about Bup-nal described experiences shaped by unmet expectations. For those that discontinued, some expected rapid pain relief similar to full-agonist opioids and felt frustrated by bup nal’s gradual or incomplete effect. Others expressed concern about daily dependence, side effects, or emotional blunting.

*“Other medicines worked faster. I didn’t like it.”*

Participant-018 (not receiving bup-nal)

*“Even taking it three times a day, I still have pain in between doses.”*

Participant-014 (receiving bup-nal)

*“Great when I’m not in that much pain. Not good for terrible crisis.”*

Participant-017 (receiving bup-nal)

Some participants also attributed treatment discontinuation to side effects.

*“It made me very nauseous. It made my stomach hurt really bad. I couldn’t keep anything down. It made me really weak. I couldn’t get up and do things and function”*

Participant-026 (not receiving bup-nal)

For these participants, bup-nal did not change the level of pain interference. Instead, pain continued to dominate decisions, emotions, and functioning, reinforcing that the medication is not a universal answer for everyone’s lived experience.

## 4. Theoretical Framework

Using the principal phenomenological themes, we developed an interpretive framework to illustrate how adults with SCD experience chronic pain and treatment (COT and bup-nal) in daily life (Figure 1). Figure 1 was developed inductively from the phenomenological themes and participant narratives identified during analysis. Concerns related to treatment effectiveness (“Will it Work?”), stigma, and uncertainty regarding pain relief emerged consistently across interviews as central influences on decisions to initiate, continue, or decline bup–nal therapy. Across narratives, participants described living in bodies where pain is constant and normalized, shaping identity, emotions, relationships, and daily functioning. Patients often structured their routines around their medications although it did not always provide sustained relief. At the same time, they felt that stigma and moral judgment complicated their care and treatment decisions. Within this context, bup-nal was experienced as a positive transformation in how pain was managed and incorporated into daily life. For some, bup-nal reduced the pain interference, enabling greater stability, function, and participation. For others, unmet expectations for acute pain crisis relief, side effects, or concerns about daily medication use meant that pain remained dominant and unresolved. The framework highlights that the key distinction across narratives was not the presence or absence of pain, but whether pain continued to dominate and disable life versus becoming more manageable and no longer dictating daily functioning, a shift most commonly described by participants receiving bup-nal.

## 5. Discussion

In this phenomenological study, adults with SCD described chronic pain as a lived experience that shaped daily life, identity, emotional well-being, relationships, and treatment decisions. Across narratives, pain was experienced as constant condition that structured how participants lived in their bodies, interacted with the world and navigated the healthcare system. Participants’ descriptions of SCD pain as qualitatively distinct, immediately recognizable, and emotionally alarming are consistent with prior qualitative studies documenting the unique sensory and affective dimensions of SCD-related-pain [33,34,35]. The combination of physical discomfort, fear, and urgency described by participants underscores that SCD pain is both nociceptive and emotional. While participants differed in whether they initiated, continued, or declined bup-nal, these differences often reflected variations in expectations, priorities, and lived experiences of pain rather than fundamentally distinct perspectives. Together, these findings offer important insights into how people with SCD and chronic pain understand pain management and how bup-nal may reshape but not eliminate the experience of pain. Similarly, these findings reinforce calls to reframe pain management goals in SCD toward functional outcomes rather than pain elimination.

Participants in this study represented a socioeconomically diverse young adult population, with relatively high levels of educational attainment but limited economic stability. Despite many having some college education, a substantial proportion reported low income and reliance on disability support. This pattern reflects a broader disconnect between educational potential and economic opportunity among adults with SCD, likely driven by the cumulative impact of chronic pain, frequent healthcare utilization, and difficulty maintaining consistent employment. This socioeconomic context is critical to understanding participants’ narratives, particularly their emphasis on treatments such as suboxone that enable daily functioning, reduce healthcare utilization, and support independence.

Stigma emerged as a powerful and consistent influence on participants’ experiences, shaping interactions with clinicians, family members, and institutions. Feelings of being judged, misunderstood, or dismissed are well documented among individuals with SCD and have been linked to delayed care-seeking, mistrust, and worse outcomes [33,36,37,38,39]. Our findings further add to this literature by demonstrating how stigma complicates both routine opioid use and perceptions of bup-nal, which participants associated with addiction treatment. The need for reassurance that bup-nal use did not equate to being labeled an “addict” highlights the intersection of racialized stigma, chronic pain, and substance use narratives. This finding is particularly salient given that buprenorphine is increasingly recognized as a potentially safer and more stabilizing option for chronic SCD pain [23,26,40], yet its association with addiction may continue to be a barrier to uptake.

A key finding of this study is that participants receiving bup-nal consistently reported meaningful improvements in daily functioning, emotional well-being, and overall life stability compared to those not receiving bup-nal. While pain was not eliminated, it was less controlling, allowing participants to re-engage with work, family, and social roles. In contrast, participants who declined or discontinued suboxone described continued experiences of pain that remained central, disruptive, and limiting across multiple domains of life. These findings align with emerging evidence from our group and others suggesting that bup-nal may reduce healthcare utilization and improved functional outcomes in adults with SCD and chronic pain [23,24,26], Importantly some participants’ noted improvement in chronic daily pain but limited efficacy during acute pain crises. This distinction (and fear) explains why some participants discontinued or declined bup-nal despite recognizing its stabilizing benefits. For these individuals, expectations for rapid relief during severe pain episodes outweighed the value of day-to-day functioning. This highlights a critical mismatch between patient expectations and the pharmacologic profile of buprenorphine and underscores the need for clear counseling and shared decision-making.

Importantly, not all participants viewed bup-nal positively or chose to continue treatment. Individuals who declined or discontinued bup-nal often described a mismatch between their treatment expectations and the medication’s effects. Many participants prioritized rapid and substantial relief of acute vaso-occlusive pain crises and perceived bup-nal as less effective than full opioid agonists for managing severe acute pain. For some, concerns regarding side effects, ongoing medication burden, or uncertainty about treatment effectiveness further contributed to dissatisfaction. Participants also expressed apprehension about losing access to full opioid agonists, particularly during episodes of severe pain, reflecting the central role that acute pain management continued to play in treatment decision-making. Consistent with previous literature describing stigma surrounding both opioid use and medications commonly associated with opioid use disorder treatment, some participants remained hesitant to initiate or continue bup-nal because of concerns about how the medication would be perceived by family members, healthcare providers, or society [41,42,43,44,45]. Together, these findings suggest that decisions regarding bup-nal are influenced not only by its effects on chronic pain and functioning, but also by expectations regarding acute pain relief, perceptions of treatment effectiveness, concerns about stigma, and individual preferences for pain management. These factors highlight the importance of shared decision-making and clear communication regarding the potential benefits and limitations of bup-nal therapy.

Although SCD genotype is associated with disease severity at the population level, pain experiences and treatment needs vary considerably among individuals with both homozygous and heterozygous forms of SCD. Importantly, adults with heterozygous genotypes may also experience severe pain, chronic complications, and substantial healthcare utilization. Because the current study focused on lived experiences of chronic pain and treatment decision-making rather than genotype-specific differences, we did not evaluate themes according to genotype. Future research should explore whether perceptions of chronic pain, opioid therapy, and bup-nal differ across SCD genotypes and levels of disease severity.

## 6. Limitations

This study has several limitations. Participants were recruited from a single comprehensive SCD center with established expertise in chronic pain management and bup-nal therapy, and which may limit the transferability of findings to other clinical settings with different resources, treatment approaches, or patient populations. Additionally, only English-speaking participants were eligible, which may limit transferability to populations with different linguistic or cultural experiences. The study used a pragmatic definition of chronic pain (pain occurring on most days for at least three months), which differs from the AAPT criteria for chronic SCD pain and may limit comparability with other studies. In addition, the sample consisted primarily of younger adults (median age 29 years), and the experiences, treatment priorities, and perceptions of bup-nal may differ among older adults living with SCD and chronic pain. The study also included a relatively small sample, consistent with qualitative research, and findings should be interpreted as providing in-depth insight into patient experiences rather than representing the views of all individuals with SCD. Participants receiving bup-nal had been on treatment for at least six months, which may have preferentially captured individuals who tolerated or benefited from treatment and may therefore underestimate experiences leading to early discontinuation. All participants were also receiving disease-modifying treatment for their SCD in addition to frequent follow-up. These experiences may differ in settings with less access to specialized care or protocols specifically designed for bup-nal therapy (such as the David Carrol protocol) [26]. Additionally, as with all qualitative research, findings reflect participants’ perceptions and may not capture all experiences. However, the depth and consistency of narratives strengthen the credibility of these findings.

## 7. Conclusions

Many adults with SCD develop chronic daily pain in addition to acute pain crisis. These adults experience pain as a constant, embodied condition that shapes daily life and treatment decisions. Participants described diverse experiences with bup-nal. While some reported improved function, greater independence, and reduced healthcare utilization, others described unmet expectations regarding acute pain relief, side effects, or concerns that contributed to treatment discontinuation or refusal. Across narratives, a central distinction emerged: participants receiving bup-nal described pain as more manageable and less disruptive to daily functioning, whereas those not on bup-nal continued to experience pain as dominant and disabling in their everyday lives. These findings suggest that successful use of bup-nal in SCD requires aligning treatment goals with patient priorities particularly emphasizing stability, function, and quality of life rather than pain elimination. Clinicians should explicitly address the distinction between chronic pain and acute crisis pain when discussing bup-nal and acknowledge that additional strategies may be needed for the management of acute pain crisis. Reducing stigma, both within healthcare settings and among families, is essential to support informed decision-making as well as patient education that reframes buprenorphine as a pain management tool rather than solely an addiction medication may facilitate acceptance. Future multicenter research is needed to evaluate the effectiveness of bup-nal compared with COT for reducing pain interference in adults with SCD using patient-centered and community-informed approaches. Such studies should prioritize outcomes that matter most to patients, including daily functioning, quality of life, healthcare utilization, and treatment burden, rather than pain intensity alone.

## Figures and Tables

**Table 1:** Demographic characteristics of study participants (*n* = 26).

Characteristic	Total Sample (*n* = 26)	Receiving Bup-Nal (*n* = 16)	Not Receiving Bup-Nal (*n* = 10)
Age, median (IQR), years	29 (23.8–31.8)	29.5 (23.0–33.8)	28.0 (26.0–30.8)
Female, *n* (%)	18 (69.2)	10 (62.5)	8 (80.0)
Male, *n* (%)	8 (30.8)	6 (37.5)	2 (20.0)
Education, *n* (%)			
High school/some college	17 (46.2)	11 (68.8)	6 (60.0)
Bachelor’s	8 (42.3)	4 (25.0)	4 (40.0)
Master’s	1 (11.5)	1 (6.2)	0 (0.0)
Annual Income, *n* (%)			
Disability income	9 (34.6)	4 (25.0)	5 (50.0)
<$25,000	6 (23.1)	5 (31.2)	1 (10.0)
$25,000–$39,000	6 (23.1)	3 (18.8)	3 (30.0)
≥$40,000	2 (7.7)	2 (12.5)	0 (0.0)
Not reported	3 (11.5)	2 (12.5)	1 (10.0)

**Table 2:** Relationship Between Codes and Phenomenological Themes.

Analytic Domain	Example Codes	Resulting Theme
Pain experience	Pain quality, fear, bodily awareness	Theme 1
Chronicity	Constant pain, adaptation	Theme 2
Daily life impact	Work, social life, planning	Theme 3
Opioid experience	Tolerance, side effects, inefficacy	Theme 4
Social context	Stigma, judgment, mistrust	Theme 5
Treatment decisions	Autonomy, expectations	Theme 6
Suboxone effects	Function, mood, hospital use	Theme 7
Non-alignment	Crisis pain, discontinuation	Theme 8

## Data Availability

De-identified excerpts of qualitative data supporting the findings of this study are included within the article. Full transcripts are not publicly available due to confidentiality considerations.
